# Neoadjuvant chemoimmunotherapy for esophageal squamous cell carcinoma with non-regional cervical lymph node metastasis: a retrospective pilot study

**DOI:** 10.3389/fimmu.2025.1611108

**Published:** 2025-09-16

**Authors:** Chufeng Zeng, Guozhen Yang, Lingyu Tan, Kun Li, Wenyu Zhai, Xin Zhang, Longgao Liu, Weihua Wu, Xiaodong Su, Jianhua Fu, Xu Zhang, Wei Wei Liu

**Affiliations:** ^1^ State Key Laboratory of Oncology in Southern China and Collaborative Innovation Center for Cancer Medicine, Department of Thoracic Surgery, Guangdong Esophageal Cancer Institute, Sun Yat-Sen University Cancer Center, Guangzhou, China; ^2^ Department of Head and Neck Surgery, Guangdong Esophageal Cancer Institute, Sun Yat-Sen University Cancer Center, Guangzhou, China

**Keywords:** esophageal squamous cell carcinoma, cervical lymph node metastasis, neoadjuvant chemoimmunotherapy, surgical resection, survival outcomes

## Abstract

**Background:**

Esophageal squamous cell carcinoma (ESCC) with non-regional cervical lymph node metastasis (CLNM) poses significant therapeutic challenges due to lack of consensus in guidelines and poor outcomes associated with conventional treatment modalities. Recent studies have demonstrated promising efficacy of combined immunotherapy, chemotherapy, and surgery in ESCC; however, the role of this multimodal approach in managing non-regional CLNM—historically considered inoperable—remains unclear.

**Methods:**

This retrospective cohort study included 15 patients with thoracic ESCC and non-regional CLNM who underwent neoadjuvant chemoimmunotherapy (nCIT), followed by McKeown esophagectomy with three-field lymphadenectomy between 2020 and 2024. CLNM was confirmed via ultrasound-guided biopsy. Data on pathological response, safety, and survival outcomes were collected and analyzed. Survival analysis was performed using the Kaplan-Meier method.

**Results:**

A pathological complete response (pCR) in CLNM was achieved in 93.3% of patients, while the Total pCR (ypT0N0M0) rate (clearance of both primary tumor and metastatic lymph node) was 33.3%. At a median follow-up of 18.0 months, the 1-year disease-free survival (DFS) rate was 91.7%. One patient died during the follow-up period. Postoperative complications occurred in 73.3% of patients, predominantly respiratory events such as atelectasis and pneumonia; only one patient experienced a grade 4 event. Treatment-related adverse events (TRAEs) were mild, with no grade ≥3 TRAEs observed; anemia was the most common TRAEs, occurring in 46.7% of patients.

**Conclusion:**

nCIT induces a high cervical nodal response in ESCC with non-regional CLNM and may redefine surgical eligibility for patients with non-regional metastases. The observed 1-year DFS of 91.7% is promising, though long-term outcomes require further validation through prospective studies.

## Introduction

Esophageal cancer remains a major global health concern due to its aggressive biological behavior and poor prognosis (1). Among its histological subtypes, esophageal squamous cell carcinoma (ESCC) shows a distinct geographical distribution, accounting for 85.8% of all esophageal cancer cases in China (2). A key clinical challenge in managing ESCC is its early tendency toward lymphatic spread. Notably, approximately 10%-20% of patients present with cervical lymph node metastases (CLNM) at the time of diagnosis (3, 4). These nodes, especially the lower deep cervical lymph nodes, serve as dispensable relay stations, may represent the final checkpoints for esophageal lymphatic drainage before tumor cells enter the venous circulation (5). Therefore, this metastatic pattern is often considered an advanced disease stage at presentation and is associated with unfavorable clinical outcomes.

The therapeutic approach to ESCC with CLNM remains a subject of ongoing debate. While major international staging systems—namely, the 8th edition of the American Joint Committee on Cancer/Union for International Cancer Control (AJCC/UICC) TNM classification (6) and the 12th edition of the Japanese Esophageal Society (JES) criteria (7)—concur in classifying cervical lymph nodes outside the paraesophageal region, particularly supraclavicular lymph nodes, as sites of non-regional metastases, differences emerge in the corresponding treatment recommendations.

The National Comprehensive Cancer Network (NCCN) guidelines recommend systemic therapy as the primary treatment modality for this subgroup (8). Conversely, the JES guidelines (9, 10) adopt a more nuanced, stage-specific approach. Based on the Japan guideline, non-regional cervical lymph node of thoracic esophageal cancer are cervical lymph nodes apart from cervical paraesophageal lymph nodes (which are generally consistent in location with the cervical level VI region). As For patients with non-regional CLNM (excluding supraclavicular nodes) or those with T4 disease and supraclavicular CLNM, chemoradiotherapy is recommended. For patients with T1-T3 tumors and supraclavicular CLNM, perioperative chemotherapy or preoperative chemoradiotherapy is suggested.

Until recent years, our center had still been treating most ESCC patients with non-regional CLNM with definitive chemoradiotherapy that demonstrated limited efficacy in the management of non-regional CLNM. A cohort analyses report a dismal 5-year overall survival (OS) rate of only 12.6% in patients with supraclavicular CLNM treated with chemoradiotherapy alone (11). This suboptimal outcome is closely associated with high rates of locoregional failure, as 61.5% of patients experience disease progression at the primary site following treatment (12), highlighting the urgent need for integrated multimodal therapeutic strategies.

In patients with thoracic ESCC and supraclavicular CLNM, neoadjuvant chemotherapy has achieved a median progression-free survival (PFS) of up to 24 months (13, 14). Neoadjuvant chemoradiotherapy (nCRT) has demonstrated improved pathological response rates, with a pathological complete response (pCR) rate of 26.2% and a 2-year OS rate of 68.2% (15). However, nCRT may be associated with increased postoperative morbidity, particularly pulmonary complications and anastomotic leakage, underscoring the need for careful preoperative risk assessment.

The advent of programmed death protein 1 (PD-1) inhibitors has transformed the treatment landscape for both advanced and locally advanced ESCC. As to advanced stage ESCC, the combination of immunotherapy and chemotherapy has been shown to extend survival (16). As to locally advanced ESCC, emerging phase II trials have highlighted the potential of neoadjuvant chemoimmunotherapy (nCIT) in improving treatment responses (17, 18). Accordingly, we hypothesized that nCIT would enhance treatment response via immune microenvironment remodeling in patients with ESCC and non-regional CLNM. Additionally, given the high local recurrence rates associated with chemoimmunotherapy, nCIT may provide added benefit for ESCC patients with non-regional CLNM by reducing recurrence risk.

In summary, current guidelines recommend systemic therapy as the standard of care for ESCC with non-regional CLNM; however, this approach is often associated with high recurrence rate. Retrospective studies suggested that combining neoadjuvant therapy with surgical resection may confer survival benefits by reducing recurrence in select patients. Nevertheless, the efficacy and safety of surgery in conjunction with modern immunochemotherapy for non-regional CLNM remain insufficiently studied. To address these gaps, this study retrospectively analyzes data from a single-center cohort of ESCC patients with non-regional CLNM who received nCIT, followed by surgery. The objective is to evaluate the impact of this multimodal strategy on survival outcomes, recurrence patterns, and treatment safety, thereby informing the optimization of therapeutic protocols for this high-risk population.

## Materials and methods

### Study population and ethical approval

This retrospective cohort study was approved by the Institutional Review Board of Sun Yat-sen University Cancer Center. Inclusion criteria consisted of histologically confirmed ESCC with CLNM (non-regional CLNM confirmed by fine-needle aspiration or core needle biopsy), completion of nCIT, and subsequent McKeown esophagectomy with standardized three-field lymphadenectomy encompassing cervical, mediastinal, and abdominal lymph nodes dissection. The included neoadjuvant regimen must consist of two chemotherapeutic agents with different mechanisms and one PD-1 inhibitor for 2–4 cycles.

Exclusion criteria included: (1) presence of synchronous or metachronous malignancies; (2) distant metastases beyond cervical lymph nodes; (3) surgical approaches other than McKeown esophagectomy; (4) non-squamous histology subtypes (e.g., adenocarcinoma, small cell carcinoma); and (5) absence of histopathological confirmation of CLNM prior to treatment.

### Pretreatment evaluation

All patients underwent comprehensive cervical ultrasonography to evaluate CLNM. Lymph nodes suspicious for malignancy—defined by a short-axis diameter ≥10 mm, round morphology, or loss of fatty hilum—were subjected to ultrasound-guided percutaneous biopsy. Histopathological confirmation was performed subsequently.

### Surgical protocol

All McKeown esophagectomies were performed by board-certified thoracic surgeons with a minimum annual volume of 50 esophagectomies. Key cervical lymphadenectomy included at least en bloc resection of bilateral cervical levels VI and IV. Depending on preoperative imaging and intraoperative findings, the extent of lymphadenectomy was expanded as clinically indicated.

### Pathological assessment

Resected specimens were evaluated in modified Mandard criteria. Complete response (CR) was defined as absence of viable tumor cells, major pathological response (MPR) was defined as ≤& 10% residual viable tumor and partial response as 10–50%; no significant response as > 50%.

### Adverse event monitoring

Treatment-related adverse events (TRAEs) were retrospectively reviewed using Common Terminology Criteria for Adverse Events (CTCAE v5.0) based on clinical records, blood tests and examinations majorly including CT scans and electrocardiograms Postoperative complications were classified according to the Clavien-Dindo system.

### Data collection and follow-up

Clinicopathological variables were obtained from prospectively maintained institutional databases, including tumor location, clinical TNM stage (UICC/AJCC 8th edition), number of treatment cycles, pathological response, receipt of adjuvant therapy, OS, and disease-free survival (DFS). Classification of treatment related adverse events followed Common Terminology Criteria for Adverse Events (CTCAE) v5.0 (19). Postoperative complications were classified according to the Clavien-Dindo grading system (20). All surgical patients were scheduled for follow-up every three months during the first postoperative year, including contrast-enhanced CT/MRI evaluations. Patients lost to follow-up were contacted via telephone, either directly or through designated proxies.

### Statistical analysis

Collected data were summarized using frequencies and percentages for categorical variables and mean values with ranges or standard deviation (SD) for continuous variables, as appropriate. The Mann-Whitney U test was used for non-parametric comparisons of continuous variables. Categorical variables were compared using the Chi-square test or Fisher’s exact test, as appropriate. Survival analysis was performed using the Kaplan Meier method based on follow-up records. All statistical analyses were conducted using R software(4.4.2).

## Results

### Baseline characteristics

A total of 15 ESCC patients with CLNM underwent McKeown minimally invasive thoracoscopic esophagectomy with three-field lymphadenectomy between January 2020 and November 2024 (**Table 1**). All patients underwent biopsy with histopathological confirmation of CLNM and subsequently received nCIT. The cohort included 12 males (80.0%), and 2 patients (13.3%) were aged ≥70 years. Pretreatment localization of CLNM involved cervical level IV in all patients (100%), level VI in 5 patients (33.3%), and level V in 1 patient (6.7%). The mean long-axis and short-axis diameters of the largest cervical lymph node were 15.2 ± 5.6 mm and 10.0 ± 3.0 mm, respectively. Regarding neoadjuvant regimens, the majority received camrelizumab-based therapy (n=13). Among these, seven patients (46.7%) received 3 cycles of camrelizumab (200 mg intravenously, day 1) in combination with nab-paclitaxel (240 mg/m² intravenously, day 1) and S-1 (40–60 mg orally twice daily based on body surface area). Five patients (33.3%) completed 4 cycles of the same regimen, and one patient (6.7%) completed 2 cycles. Two patients received alternative regimens: one received 2 cycles of tislelizumab (200 mg intravenously, day 1) with nab-paclitaxel and nedaplatin (80 mg/m² intravenously, day 1), and the other completed 3 cycles of sintilimab (200 mg intravenously, day 1) combined with nab-paclitaxel and cisplatin (75 mg/m² intravenously, day 1).

**Table 1:** Characteristic of neoadjuvant chemoimmunotherapy for patients with non-regional CLNM.

Variable	Non-regional CLNM (n=15)
Age ≥70 years, n (%)	2 (13.3%)
Gender, n (%)	
Male	12 (80.0%)
Female	3 (20.0%)
Tumor location, n (%)	
Upper Third	1 (6.7%)
Middle Third	11 (73.3%)
Lower Third	3 (20.0%)
Baseline CLNM distribution, n (%)	
Level VI	5 (33.3%)
Level V	1 (6.7%)
Level IV	15 (100.0%)
Bilateral CLNM, n (%)	4 (26.7%)
Baseline CLNM amount, n (%)	
1	3 (20.0%)
>1	12 (80.0%)
Largest CLN measurements, Mean ± SD (mm)	
Long-axis diameter	15.2 ± 5.6
Short-axis diameter	10.0 ± 3.0
Clinical T stage, n (%)	
2	3 (20.0%)
3	12 (80.0%)
Clinical N stage, n (%)	
1	5 (33.3%)
2	9 (60.0%)
3	1 (6.7%)
Neoadjuvant cycles, n (%)	
2	2 (13.3%)
3	8 (53.3%)
4	5 (33.3%)
Adjuvant Therapy, n (%)	7 (46.7%)

n (%) Amount (proportion); SD, standard deviation; CLNM, cervical lymph node metastasis; CLN, cervical lymph node.

### Efficacy

All surgical procedures achieved R0 resection. The median operative time was 400.0 ± 246.6 minutes. The median number of lymph nodes harvested was 64.0 ± 21.9 per specimen, including a median of 17.0 ± 10.8 cervical lymph nodes. Postoperative recovery metrics showed a median intensive care unit (ICU) stay of 2.0 ± 2.5 days and a median total hospitalization duration of 16.0 ± 8.6 days (**Table 2**).

**Table 2:** Major outcomes of neoadjuvant chemoimmunotherapy for patients with non-regional CLNM.

Outcomes	Non-regional CLNM (n=15)
Pathological T stage, n (%)	
0	5 (33.3%)
1	4 (26.7%)
2	3 (20.0%)
3	3 (20.0%)
Pathological N stage, n (%)	
0	11 (73.3%)
1	3 (20.0%)
2	1 (6.7%)
Pathological M stage = 1, n (%)	1 (6.7%)
Pathological stage, n (%)	
I	9 (60.0%)
II	1 (6.7%)
III	4 (26.7%)
IV	1 (6.7%)
Surgical length, Median ± SD (minutes)	400.0 ± 246.6
Dissected CLNM amount, Median ± SD	17.0 ± 10.8
Total dissected lymph nodes, Median ± SD	64.0 ± 21.9
CLNM pCR, n (%)	14 (93.3%)
Total pCR, n (%)	5 (33.3%)
Total MPR, n (%)	8 (53.3%)
ICU length of stay (day), Median ± SD	2.0 ± 2.5
Hospital length of stay (day), Median ± SD	16.0 ± 8.6

n (%) Amount (proportion); SD, standard deviation; CLNM, cervical lymph node metastasis; CLN, cervical lymph node; pCR, pathologic complete response; MPR, major pathologic response; ICU, intensive care unit.

The pathological TNM staging was classified as pTNM according to the 8th edition of the TNM classification for esophageal cancer.

Pathological assessment revealed a substantial treatment response in both the primary tumor and CLNM. Among the 15 patients, 93.3% (14/15) achieved complete eradication of metastatic disease in all dissected cervical lymph nodes. A pCR (ypT0) in the primary tumor was observed in 33.3% (5/15) of patients, all of whom also achieved ypN0M0 status. Additionally, 53.3% (8/15) demonstrated an MPR.

**Figure 1:**
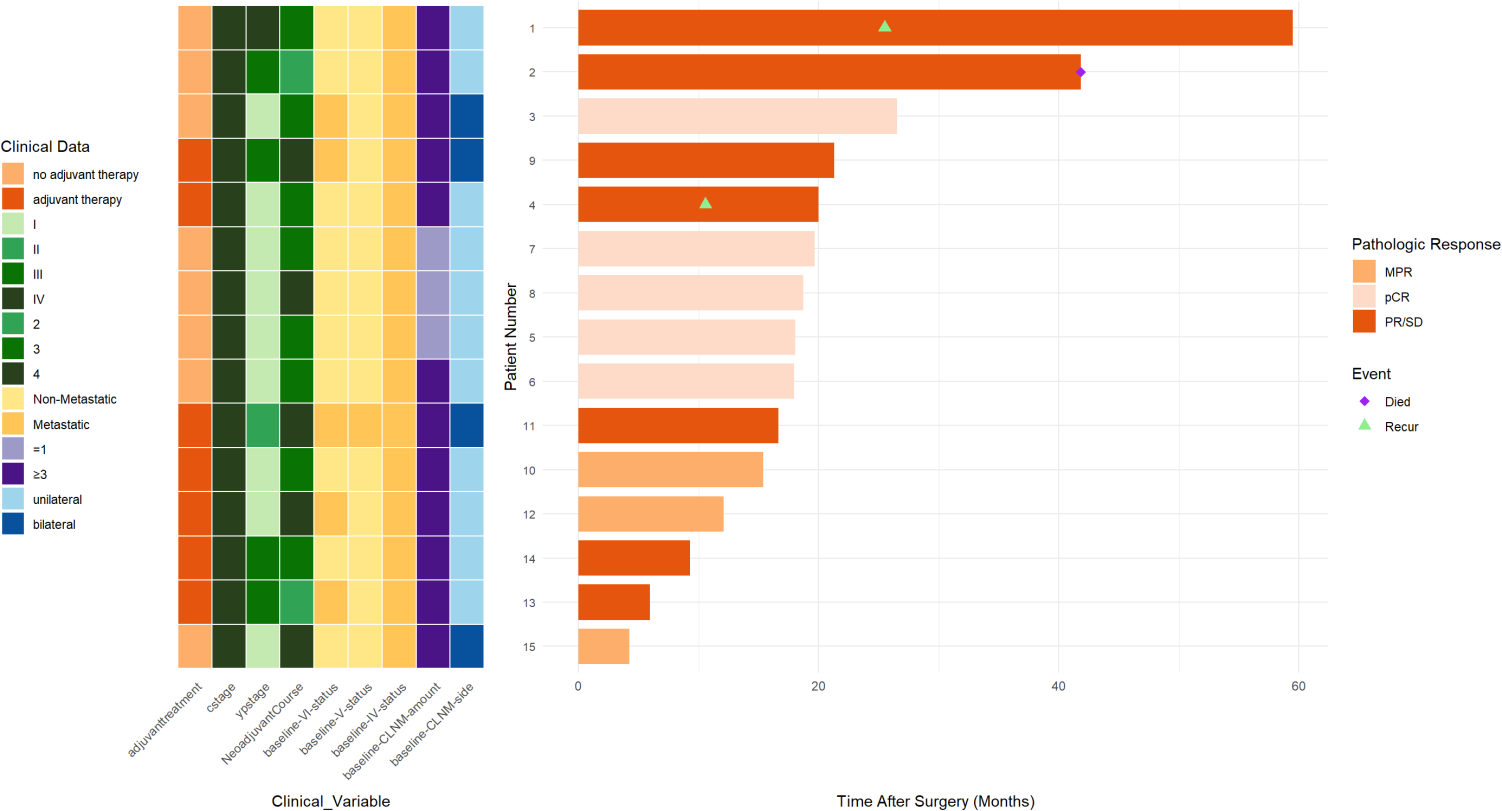
Swimmer plot depicting tumor response to neoadjuvant therapy and follow-up. pCR, pathologic complete response; MPR, major pathological response; PR/SD, partial response or stable disease; CLNM, cervical lymph node metastasis; cstage, clinical stage; ypstage, post-neoadjuvant therapy pathological stage.

**Figure 2:**
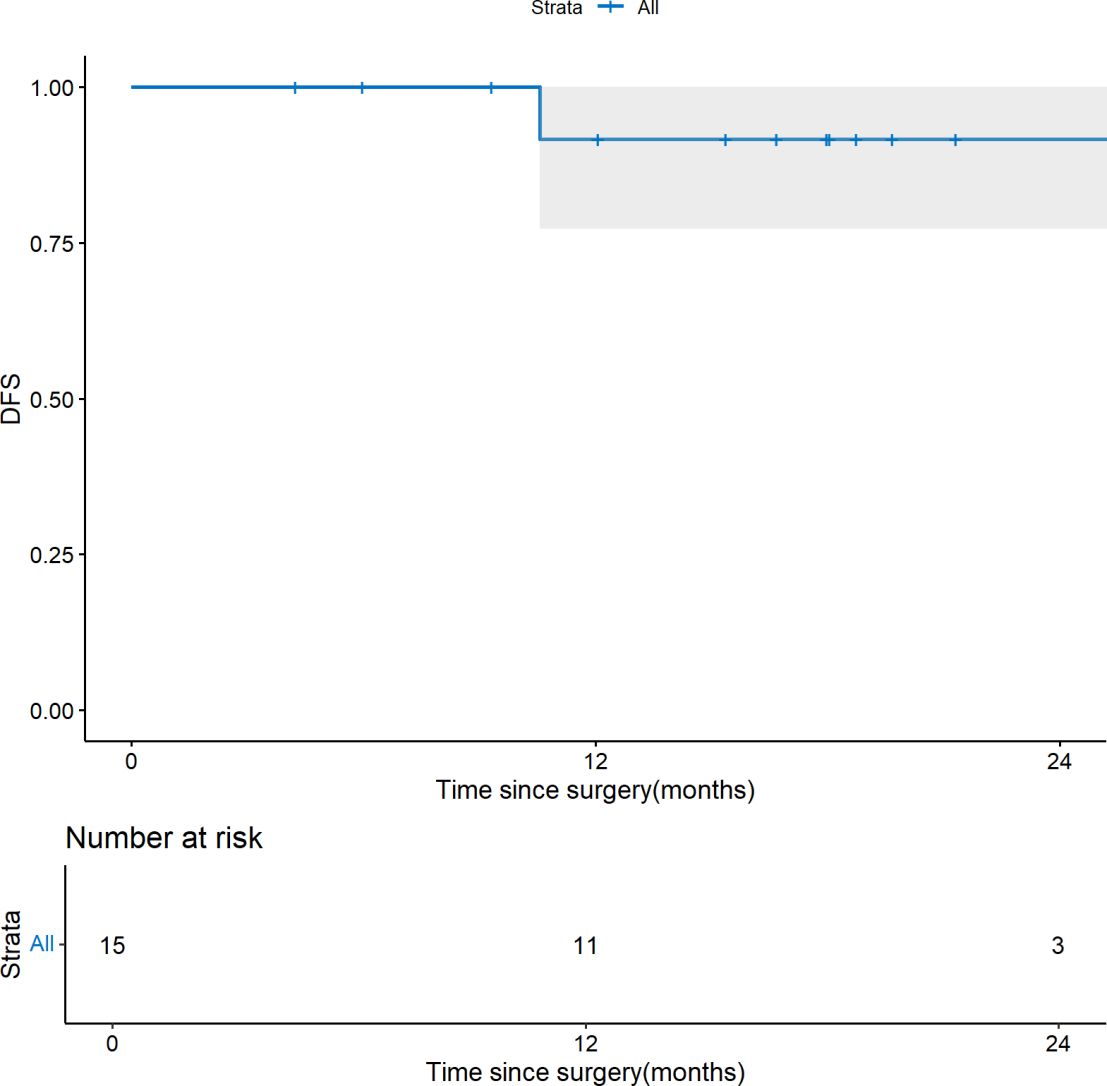
Kaplan-Meier survival curve showing disease-free survival (DFS).

Details regarding survival outcomes, pathological response, and associated clinical characteristics are illustrated in **Figure 1**. At a median follow-up of 18.0 months (interquartile range [IQR]: 13.7–20.7), the cohort exhibited a 1-year DFS rate of 91.7% (95% CI 77.3%-100%). One patient died of non-tumor-related causes 41.8 months after esophageal cancer surgery without recurrence. Tumor recurrence occurred in two patients, both involving the mediastinum and cervical lymph nodes. Notably, both of these patients had baseline CLNM limited to level IV. One of them had a post-treatment pathology of ypT3N0M1, with residual cancer identified in the dissected cervical lymph nodes; the other had an ypT2N0M0 pathology. The Kaplan-Meier survival curve is presented in **Figure 2**.

**Figure 3:**
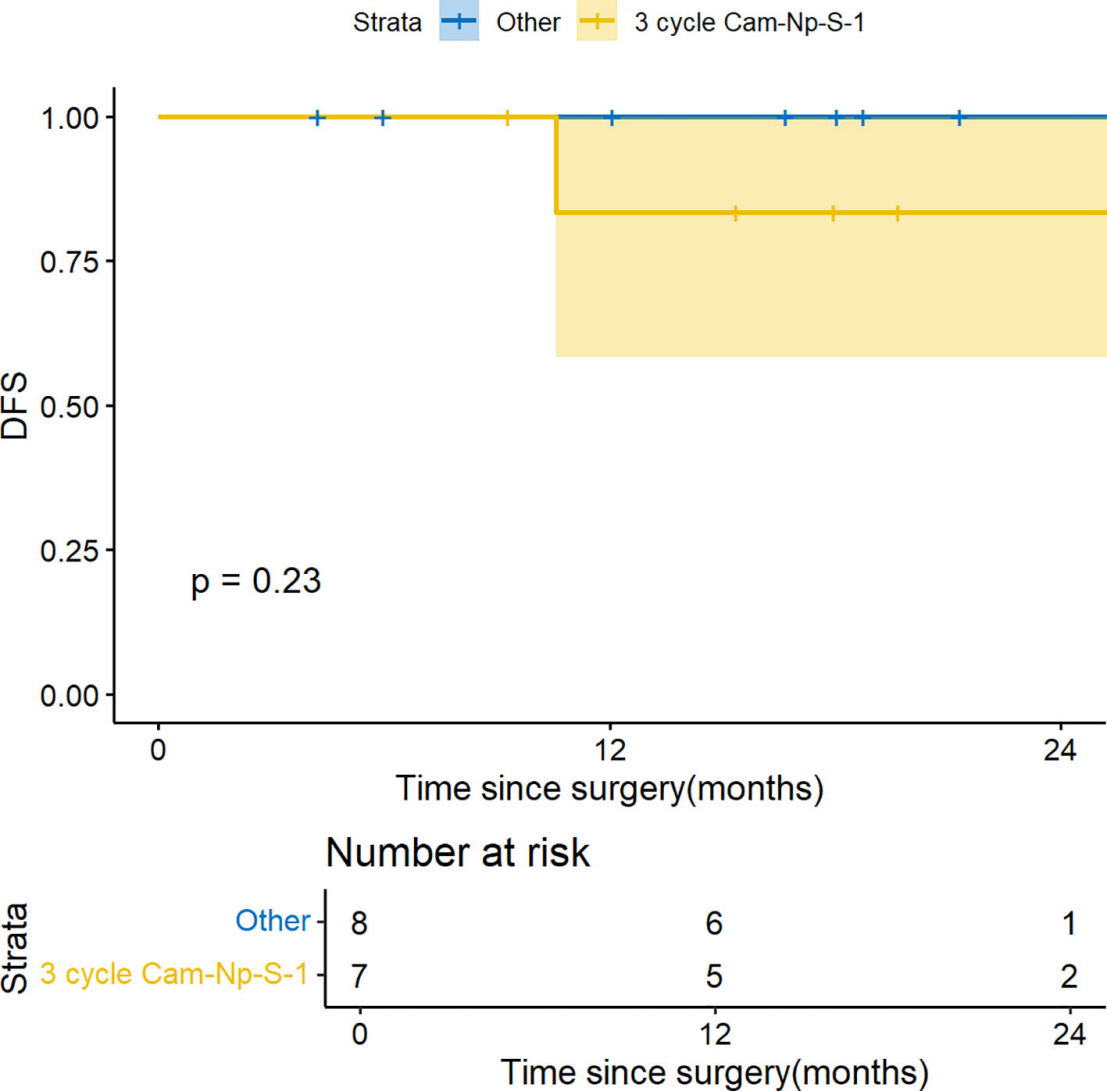
Kaplan-Meier survival curve: sensitivity analysis of 3 cycles regimen of camrelizumab, nab-paclitaxel and S-1.

As an exploratory sensitivity analysis, we divided these patients into two groups: one receiving the most frequently administered 3 cycles of camrelizumab, nab-paclitaxel and S-1 and the other receiving the remaining therapies, for comparison to test the robustness of the results. One-year DFS remained consistent (83.8% vs. 100%, p = 0.23) (**Figure 3**), and total pCR (ypT0N0M0) rate were similar (42.9% vs. 25%, p = 0.85). Due to the small sample size, these findings should be interpreted cautiously.

### Subgroup analysis: pathological response of primary tumor and metastatic lymph nodes to nCIT in ESCC

**Figure 4:**
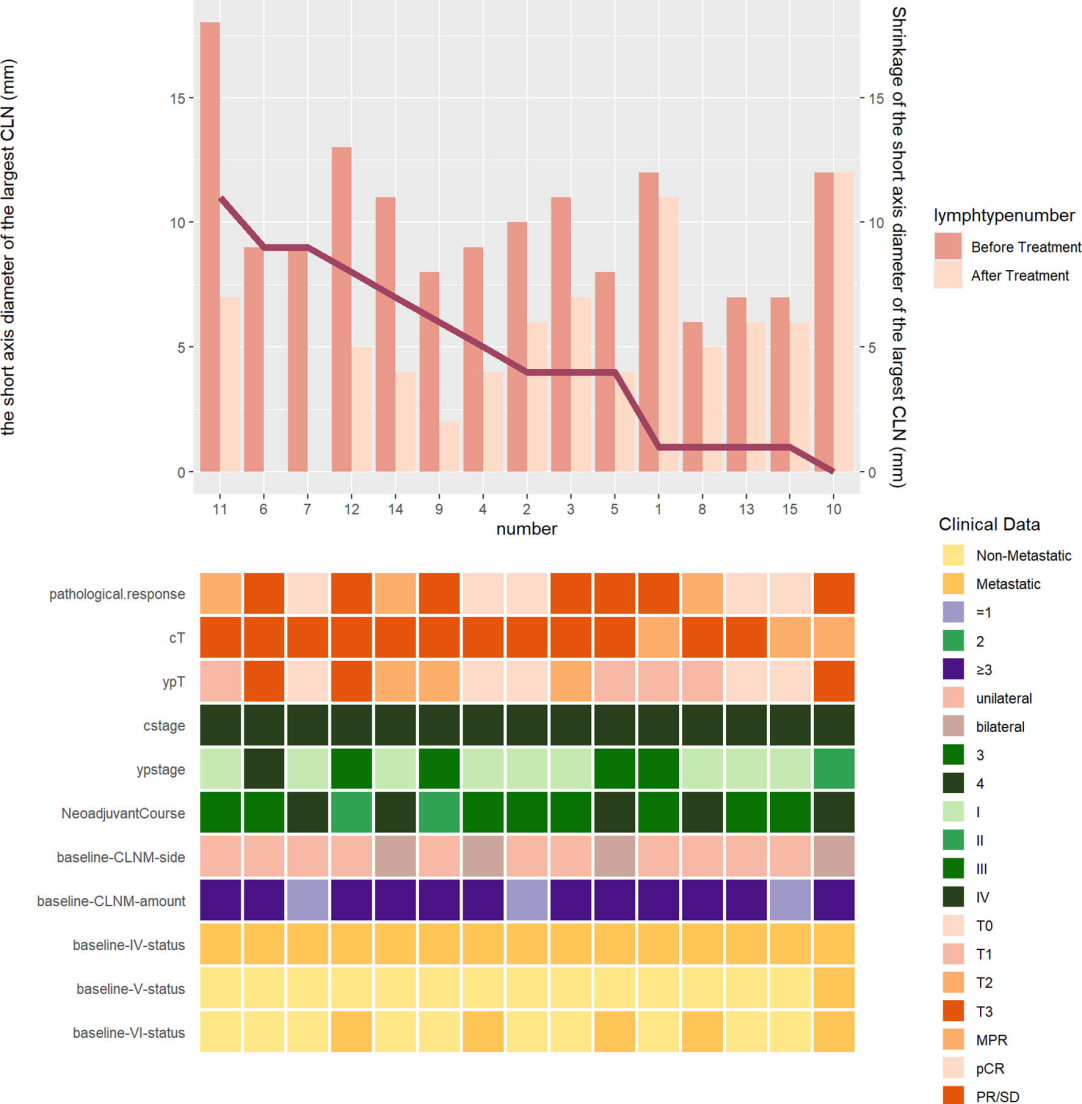
Ultrasound assessment of CLNM short-axis diameter shrinkage before and after neoadjuvant therapy in esophageal cancer. CLNM, cervical lymph node metastasis; cstage, clinical stage; cT, clinical T stage; ypstage, post-neoadjuvant therapy pathological stage; ypT, post-neoadjuvant therapy pathological T stage; ypN, post-neoadjuvant therapy pathological N stage.

As illustrated in **Figure 4**, the short-axis diameter of the largest CLN showed a notable mean reduction of 4.7 ± 3.5 mm following neoadjuvant treatment. The mean reduction in long-axis diameter was 6.2 ± 6.2 mm. Notably, in two patients, ultrasound evaluation after nCIT revealed no evidence of metastatic lymph nodes corresponding to baseline. No significant difference was observed in pCR rates between the primary lesion (5/15) and CLNM (14/15) (p>0.999).

However, heterogeneity in lymph node response was observed. Among the 14 patients who achieved cervical lymph node clearance, four were pathologically staged as ypN+ due to residual disease in thoracic or abdomen regional lymph nodes. These patients did not experience recurrence during the follow-up period.

Furthermore, the number of neoadjuvant therapy cycles (2–3 vs. 4 cycles) showed no statistically significant association with cervical lymph node pCR (p>0.999) or primary tumor pCR (p=0.600).

### Safety

Nine patients (60%) had TRAEs (**Table 3**). No grade ≥3 TRAEs were observed. The most common TRAEs were anemia (46.7%), followed by arrhythmia (13.3%), abnormal liver function (6.7%), hypothyroidism (6.7%), and leukopenia (6.7%). All TRAEs were classified as grade 1–2. No treatment discontinuations or fatal events were attributable to nCIT.

**Table 3:** Treatment-related adverse events (TRAEs).

TRAEs, *n* (%)	Grade 1–2
Anemia	7 (46.7%)
Arrhythmia	2 (13.3%)
Abnormal liver function	1 (6.7%)
Hypothyroidism	1 (6.7%)
Leukopenia	1 (6.7%)

Number (percentage).

Importantly, no 90-day mortality was reported. Postoperative complications occurred in 73.3% (11/15) of patients, with respiratory complications being the most prevalent (**Table 4**). The most frequent complication was atelectasis (46.7%), followed by pulmonary dysfunction (33.3%), pneumonia (33.3%), and pneumothorax that required intervention (20.0%). The severity of postoperative complications is presented in **Table 4**. Nine patients (60.0%) experience CD 3-4 postoperative complications, and only one patient (6.7%) experienced a grade 4 complication (pulmonary dysfunction).

**Table 4:** Postoperative complications (N = 15).

Postoperative complications, n (%)	CD 1-2	CD 3	CD 4
Atelectasis	7 (46.7)	0	0
Pulmonary disfunction	0	4 (26.7)	1 (6.7)
Pneumonia	5 (33.3)	0	0
Anastomotic stenosis	0	3 (20.0)	0
Pneumothorax requiring intervention	0	3 (20.0)	0
Recurrent nerve injury	3 (20.0)	0	0
Anastomotic leakage	3 (20.0)	0	0
Abnormal liver function	1 (6.7)	0	0
Arrythmia	0	0	0
Chyle leak	0	0	0

CD, Clavien-Dindo postoperative complication classification.

Number (percentage).

## Discussion

Our investigation demonstrates that nCIT induces substantial pathological responses in thoracic ESCC with non-regional CLNM, achieving a pCR rate of 93.3% in CLNM, a pCR of 33.3% in primary tumor (ypT0). The cohort exhibited exceptional 1-year DFS rates of 91.7%, a finding that challenges conventional survival expectations for this advanced-stage population. These results align with recent evidence suggesting that nCIT may enhance survival outcomes in esophageal malignancies. The observed therapeutic benefit likely arises from the dual action of nCIT, which not only facilitates tumor debulking but also modulates the immune microenvironment.

The remarkable cervical nodal response rate observed in this pilot study of nCIT for ESCC with CLNM provides compelling insights into the potential synergy between chemotherapy and immune checkpoint inhibitors (ICIs) in modulating the tumor microenvironment within metastatic lymph nodes. This response may be partly attributable to the heightened immunogenicity of lymph nodes relative to primary tumors, as reported by Xu et al. (21). Lymph node metastases have been shown to exhibit higher tumor mutational burden (TMB) and elevated programmed cell death ligand 1 (PD-L1) expression compared to corresponding primary lesions, potentially enhancing their susceptibility to ICIs (21, 22). Moreover, lymph node-specific immune mechanisms, such as enhanced antigen presentation and enriched T cell infiltration, may further contribute to this differential response. Studies have revealed that the lymph node environment induces an IFN−γ–mediated state in metastatic cancer cells, promoting MHC−II expression and local regulatory T−cell expansion, highlighting a unique immune−modulatory niche in lymph nodes compared to primary tumors (23).

In our analysis, four of 14 patients who achieved pCR in CLNM were found to have residual tumor in other regional lymph nodes. This differential nodal response to neoadjuvant therapy may reflect underlying tumor heterogeneity and microenvironmental influences. As demonstrated by Glaeser et al. (24) in a similar study on lymph nodes response heterogeneity in breast cancer, variations in treatment response may correlate with distinct cancer subtypes. Intratumoral genetic diversity could result in subclones harboring intrinsic resistance mechanisms, such as mutations conferring reduced drug sensitivity or enhanced survival signaling. We hypothesize that anatomical location alone is unlikely to explain this effect; instead, it reflects underlying spatial heterogeneity (25). Further studies are warranted to elucidate the mechanisms underlying heterogenous lymph node response and to guide personalized therapeutic strategies.

From a clinical standpoint, the observed nodal response rate challenges traditional paradigms in ESCC management. Current guidelines frequently regard non-regional CLNM—particularly supraclavicular metastases—as indicative of advanced disease, often limiting patients to non-surgical systemic therapy. However, our findings suggest that nCIT may effectively ‘downstage’ these cases, potentially redefining the boundary between locally advanced and metastatic ESCC. This raises the compelling possibility of curative-intent surgery in patients previously considered inoperable, although extended follow-up is necessary to determine whether nodal response confers long-term survival benefits. Importantly, the observed discrepancy between complete response in primary tumors and metastatic lymph nodes underscores the need to further investigate organ-specific microenvironmental factors that may influence treatment efficacy.

Regarding survival outcomes, only one death was recorded during the follow-up period and the patients had no disease recurrence The 1-year DFS rate of 91.7% observed in our cohort of patients with non-regional CLNM represents a favorable outcome, challenging established prognostic expectations for this advanced-stage population. In comparison, previous studies reported a 1-year and 5-year OS of 56.1% and 12.6%, respectively, for patients treated with definitive chemoradiotherapy in the setting of supraclavicular CLNM (11). Similarly, neoadjuvant chemotherapy alone yielded a 1-year PFS rate of approximately 85%, as reported by Yu et al. (13). Our findings not only highlight the potential of nCIT to render previously unresectable disease amenable to surgery, but also prompt important discussions on the immunological and clinical significance of the observed high DFS rate.

Regarding recurrence patterns, although systemic therapy remains the predominant treatment paradigm, retrospective studies have reported high local recurrence rate of up to 61.5% following such regimens (12, 26). In contrast, our study demonstrated a 1-year DFS rate of 91.7% among patients with non-regional CLNM who received nCIT followed by surgery, with a notably low local recurrence rate of 13.3% (2/15). This survival advantage may be attributed to several synergistic mechanisms. Surgical resection can eliminate residual micrometastases that are resistant to systemic therapy—particularly within nodal basins where immunotherapy efficacy may be attenuated by fibrotic or immunosuppressive microenvironments post-treatment (27). Furthermore, achieving an R0 resection may reduce TMB, potentially limiting the evolution of resistant subclones that drive distant recurrence. These findings challenge the prevailing notion that non-regional CLNM invariably indicates systemic incurability and instead support the integration of surgery as a potentially curative component within a multimodal treatment framework.

Furthermore, the safety profile of nCIT in ESCC with non-regional CLNM represents a critical advancement in balancing therapeutic efficacy with tolerability. Our study demonstrates that the integration of PD-1 inhibitors with chemotherapy yields a manageable toxicity spectrum. All TRAEs were classified as grade 1–2 per CTCAE v5.0 criteria, with no grade ≥3 events or treatment discontinuations observed. The most common toxicities—anemia (46.7%) and arrhythmia (13.3%)—were primarily attributable to chemotherapy agents rather than immunotherapy. Postoperative complications occurred in 73.3% of patients, aligning with expectations for esophagectomy. The most common events included atelectasis, pulmonary dysfunction, and pneumonia. Importantly, only one Clavien-Dindo grade 4 complication (Pulmonary disfunction) was documented, and it was not associated with immunotherapy. Anastomotic leakage was observed in three patients (20.0%), and no chyle leaks were reported. Postoperative complications seemed frequent in the cohort, and this might be attributable to regimen of neoadjuvant chemo-immunotherapy, which can alter tissue planes and increase operative difficulty, followed by a radical esophagectomy. Additionally, the procedure included a three-field lymphadenectomy, which is intrinsically associated with higher risks of pulmonary complications and recurrent laryngeal nerve injury due to the extensive dissection in the neck and superior mediastinum. However, every complication, including the most severe (Grade 4 pulmonary dysfunction), was promptly identified and managed through standardized protocols and the Grade 3 complications were resolved effectively, causing nil postoperative death. No postoperative death occurred. These findings underscore the necessity for enhanced perioperative support, such as prehabilitation programs targeting respiratory function. While derived from a single-center experience, the consistent safety signal across heterogeneous nCIT regimens suggests class-wide tolerability. Collectively, these results support the feasibility of delivering aggressive multimodal therapy without compromising patient safety.

To further optimize patient selection and therapeutic outcomes in ESCC treated with nCIT, robust biomarker development is imperative. Although PD-L1 expression and TMB have demonstrated predictive value in advanced ESCC, their utility in the neoadjuvant setting remains limited (28, 29). Emerging biomarkers—such as the tertiary lymphoid structures density or spatial transcriptomic profiling of residual nodal disease—show promise in enhancing response prediction and identifying patients most likely to benefit from nCIT (30). Further research should prioritize the discovery and validation of such biomarkers to enhance precision in patient stratification and guide individualized treatment strategies. By integrating novel biomarkers with adaptive therapeutic frameworks, we can better leverage the potential of nCIT to improve outcomes, particularly in high-risk ESCC populations.

To the best of our knowledge, this is the first retrospective study to specifically evaluate the efficacy of nCIT in patients with ESCC and non-regional CLNM. Previous neoadjuvant studies have typically excluded this subgroup or classified them alongside patients with distant metastases. Our findings suggest that CLNM may represent an intermediate disease state—biologically distinct from distant metastases and potentially amenable to curative-intent strategies. The observed 1-year DFS rate of 91.7% supports this hypothesis and indicates substantial responsiveness of CLNM to immune-based therapies. Importantly, our study’s stringent inclusion criteria—requiring histopathological confirmation of CLNM prior to treatment—addresses a key limitation of previous investigations that relied solely on radiographic assessment. By replacing imaging-based assumptions with cytological and histological validation, our data contribute a higher level of evidentiary rigor to the evolving landscape of esophageal oncology. This approach highlights a potential therapeutic frontier in managing ESCC with nodal metastases.

Despite these encouraging findings, several limitations constrain the immediate clinical applicability of this study. The small sample size (n=15) might lead to potential Type II errors and limit the ability to draw definitive conclusions regarding the superiority of nCIT over existing standard-of-care regimens. The lack of long-term survival data also precludes assessment of sustained efficacy and potential late recurrences. Moreover, the study cohort may not adequately represent the broader ESCC population, as factors such as co-morbidity profiles, ethnic diversity, and sociodemographic variation were not comprehensively captured. These limitations underscore the need for larger, multicenter prospective trials to validate our findings.

In conclusion, this retrospective pilot study highlights the transformative potential of nCIT in redefining treatment strategies for thoracic ESCC with non-regional CLNM. The remarkable nodal response rates observed challenges long-standing prognostic paradigms and provide a novel clinical framework for exploring immune-chemotherapy synergy in lymphatic metastases. Furthermore, the high 1-year DFS rate supports reclassifying such cases as potentially curable, advocating for the integration of multimodal nCIT-based approaches. The promising efficacy observed here supports the design of prospective, randomized phase II/III trials to validate these results and control for confounders. We also advocate for biomarker-integrated studies to identify patients most likely to benefit from nCIT.

## Data Availability

The raw data supporting the conclusions of this article will be made available by the authors, without undue reservation.
